# The paradox of MRI for breast cancer screening: high-risk and dense breasts—available evidence and current practice

**DOI:** 10.1186/s13244-024-01653-4

**Published:** 2024-03-27

**Authors:** Francesco Sardanelli, Veronica Magni, Gabriele Rossini, Fleur Kilburn-Toppin, Nuala A. Healy, Fiona J. Gilbert

**Affiliations:** 1 Department of Biomedical Sciences for Health, Università degli Studi di Milano, Via Mangiagalli 31, Milano, 20133 Italy; 2 Unit of Radiology, IRCCS Policlinico San Donato, Via Morandi 30, San Donato Milanese, 20097 Italy; 3 Postgraduate School in Radiodiagnostics, Università degli Studi di Milano, Via Festa del Perdono 7, Milano, 20122 Italy; 4 Cambridge Breast Unit, Cambridge University Hospitals NHS Foundation Trust, Addenbrookes’ Hospital, Hills Road, Cambridge, UK; 5 Department of Radiology, School of Clinical Medicine, University of Cambridge, Level 5, Cambridge Biomedical Campus, Box 218, Cambridge, UK

**Keywords:** Breast neoplasms, Breast density, Mammography, Magnetic resonance imaging, Screening

## Abstract

In the mid-1990s, the identification of BRCA1/2 genes for breast cancer susceptibility led to testing breast MRI accuracy in screening women at increased risk. From 2000 onwards, ten intraindividual comparative studies showed the marked superiority of MRI: the sensitivity ranged 25−58% for mammography, 33−52% for ultrasound, 48−67% for mammography plus ultrasound, and 71−100% for MRI; specificity 93–100%, 91–98%, 89–98%, and 81–98%, respectively. Based on the available evidence, in 2006–2007, the UK National Institute for Clinical Excellence and the American Cancer Society recommended MRI screening of high-risk women, followed by other international guidelines. Despite evidence-based medicine ideally requiring randomised controlled trials (RCTs) for policy changes regarding screening procedures, breast MRI for high-risk screening was adopted in many countries worldwide. In 2019, the results of the “DENSE” RCT were published in favour of breast MRI screening of women with extremely dense breasts compared to mammography alone, showing a reduction of more than 80% of the interval cancer rate in women who attended MRI screening. Even though international recommendations in favour of this practice were issued, substantial obstacles still prevent health systems from adopting breast MRI for screening women with extremely dense breasts. A paradox is evident: we adopted a screening procedure without evidence from RCTs, and now that we have this level-1 evidence for the same procedure, we fail to do so. This critical review tries to explain the differences between the two cases, as examples of the complex pathways of translating radiological research into everyday practice.

**Critical relevance statement** The high-level evidence in favour of breast MRI screening of women with extremely dense breasts is failing to persuade policy makers to translate this into clinical practice.

**Key points**

• Breast MRI screening of high-risk women was adopted on basis of the evidence provided by test accuracy comparative studies showing an MRI performance greatly superior to that of mammography.

• Breast MRI screening of women with extremely dense breasts has not been adopted although the evidence of a large reduction in interval cancer rate from a RCT.

• We illustrate the differences between the two cases, as an example of the complex ways of translation of radiological research in clinical practice according to the EBM theory.

**Graphical Abstract:**

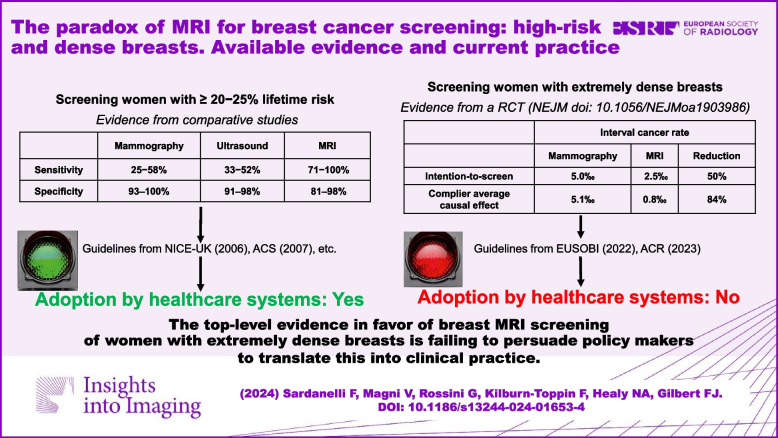

## Introduction

Breast cancer (BC) was explored with nuclear magnetic resonance (NMR) before its evolution into magnetic resonance imaging (MRI): in 1978, Goldsmith et al. [1] showed a significant difference in NMR relaxation times between benign and malignant breast tissues. In the 1970s, authors were thinking about NMR as a support system for pathologists. However, MRI became clinically available in 1981 and a revolution happened in the entire world of medical imaging.

Throughout the early 1980s, researchers explored the potential of breast MRI using the simple sequences available in those days [2–4] without injection of any contrast agent. Results were disappointing due to the large overlap of T1, T2, and proton-density values of normal and pathological tissues as well as of benign and malignant neoplasms, with the exception of normal fat and serous cysts. With the intravenous administration of a linear gadolinium-based contrast agent (the first one available, i.e. gadopentetate dimeglumine) Heywang et al. [5] began to appreciate the contrast biodistribution in breast tissues through T1-weighted images in a small group of 20 patients. The abstract states “All carcinomas enhanced, whereas dysplastic tissue enhanced slightly or not at all. […]. MR imaging of breast using Gd-DTPA may be helpful for the evaluation of dense breasts and the differentiation of dysplasia and scar tissue from carcinoma”. Other authors followed this pathway, among them the relevant group of Kaiser et al. [6].

During the same time period, there were technical developments of the breast MRI technique, including improved dedicated bilateral radiofrequency coils, better spatial resolution, T1-weighted dynamic sequences, temporal subtraction, and fat suppression/saturation [7]. Concurrently, a new emergent clinical demand arose for breast MRI after the identification of the role of the BRCA1 gene in breast and ovarian cancer susceptibility by Miki et al. in 1994 [8] and of BRCA2, a second BC susceptibility gene, by Wooster et al. in 1995 [9].

For researchers interested in innovations in BC care, these advances in knowledge and technology had opened a new scenario. They now could: (1) verify the diagnostic performance of MRI in a screening setting with a high BC incidence; (2) offer to women with hereditary predisposition to BC a possibility of an earlier detection than that offered by mammography. Notably, these high-risk women needed to be screened from a young age and were known to likely have dense breasts (with an obvious interplay between the two factors). As Heywang et al. had suggested in 1986 [5], breast density emerged as a relevant variable in the game among diagnostic modalities.

In this historical context, researchers started studies of MRI for screening high-risk women in several countries. From 2000 onwards, the publication of their results showed a large superiority of MRI versus mammography in terms of accuracy and sensitivity. After the pivotal recommendations from the National Institute for Clinical Excellence (NICE) – National Collaborating Centre for Primary Care, UK in 2006 [10] and from the American Cancer Society (ACS), USA, in 2007 [11], contrast-enhanced MRI was adopted in many countries as a screening tool for women with high hereditary BC predisposition. Variations in the adoption regarded non-negligible issues such as the level of risk and age range to undergo MRI screening or whether to perform mammography when MRI is negative [12]. For instance, considering the different age ranges for high-risk surveillance, the Department of Health in Australia recommends annual MRI surveillance for women under the age of 50 who are at high risk of developing BC [13]. In contrast, in the USA [14] and various European countries such as Austria [15], Germany [16], Italy [17], and Spain [18], the beginning of MRI surveillance is advised at the age of 25. Considering imaging modalities applied for high-risk screening, the guidelines for high-risk women in Australia [13] and Israel [19] recommend annual MRI alone, while other countries such as Austria [15], Belgium [20], Canada [21], Norway [22], Spain [18], the UK [23], and the USA [11] all indicate that MRI should be performed in adjunct to annual mammography.

The theory of evidence-based medicine (EBM), which was established in the 1990s in a clear theoretical framework by the group guided by D. Sackett [24], requires the results of RCTs to decide whether to adopt a screening test. This had been the case with population-based mammography screening from 50 to 70 years of age. A complete table about the levels of evidence needed for diagnostic tests, including their use for screening, is available at the website of Centre for EBM at the Oxford University, UK (https://www.cebm.ox.ac.uk/). However, the level of evidence needed to justify the practice of MRI screening of high-risk women was not discussed. One exception was a letter to the *British Medical Journal* by Irwig et al. in 2006, entitled “Evaluating new screening tests for breast cancer” [25], in the context of a specific debate about overdiagnosis estimated 15 years after the end of Malmö mammographic screening trial [26]. The authors [25] underlined the need of randomised controlled trials (RCTs) to assess the reduction in mortality (long-term design), to estimate overdetection/overdiagnosis (both long- and short-term design), and to compare the interval cancer rates as well as the rates of advanced cancers detected by subsequent screening rounds. In particular, they emphasised that “reducing the rate of interval cancer rates is crucial, representing the potential benefit of early detection rather than overdetection”. However, their conclusions took into account that RCTs to detect interval cancers could be considered “unnecessary or even unethical in people who are at substantially increased risk of developing cancer—for example, women at high risk of breast cancer because of gene mutations”.

This was the core of the problem: researchers did not want to randomise a BRCA1/2-mutated woman (with an individual BC lifetime risk of at least 60−70%) and risk not getting an MRI. However, on the methodological level, intraindividual sensitivity/specificity studies—those that had “cleared” breast MRI for high-risk screening by the NICE and the ACS—were theoretically a weak basis for the adoption of this screening procedure.

More than 38 years have passed since the first paper on contrast-enhanced breast MRI [5] and 16 years after the ACS recommendations for breast MRI for high-risk screening [11]. Four years ago, in 2019, unquestionable results of the “DENSE” RCT were published [27] in favour of breast MRI screening of women with extremely dense breasts, i.e. the breast density *d* class of the American College of Radiology (ACR) Breast Imaging Reporting and Data System (BI-RADS) [28]. A reduction of more than 80% of the interval cancer rate in women who attended MRI screening versus those who did not was observed. International recommendations in favour of this practice such as those from the EUSOBI [29] were issued. However, substantial obstacles still prevent health systems from adopting breast MRI for screening women with extremely dense breasts.

A paradox is evident: we adopted a screening procedure without evidence from RCTs, and now that we have evidence from RCTs for the same procedure, we fail to do so. Even in the Netherlands, where the DENSE trial has been conducted, considerable difficulties hinder its implementation [30].

This critical review aims to spotlight this issue, trying to explain the differences between the two cases, as examples of the complex pathways of translating radiological research into everyday practice.

## The conceptual EBM framework of screening tests

To understand the above-mentioned EBM conceptual framework that guides the adoption of screening tests, we take into consideration the oldest example in radiology, i.e. screening mammography. In 1998, the UK National Screening Committee defined screening as the systematic application of a test to identify apparently healthy individuals at increased risk of a specific disorder, in order to offer information, further investigation, or treatment, as appropriate [31]. As recently stated by the World Health Organization, a specific target population is invited from central records to perform simple tests to detect individuals with a disease who do not yet exhibit symptoms. Furthermore, screening programmes should be implemented only after establishing their effectiveness, ensuring adequate resources, having facilities for diagnoses, as well as for treatment and follow-up, and when the prevalence of the disease justifies the costs associated with screening. Finally, screening efforts should be justified by the considerable advantages it may offer (in terms of disease secondary prevention and disability/mortality reduction), which significantly outweigh any potential negative consequence [32]. This document clearly distinguishes between “screening” as applied to apparently healthy individuals from “early diagnosis” as applied to individuals with sign or symptoms (the “clinical” or “diagnostic” context).

In fact, screening addresses disorders that, while occurring with varying frequencies in the lives of individuals, remain consistently uncommon in each screening round, leading to a low pretest probability of disease. In the case of BC screening, this probability will be higher at the first round (“prevalent” BCs, i.e. those that developed and are detectable up to that time) and lower in later rounds (“incident” BCs, i.e. the new ones). In this scenario, a test with a sufficiently high sensitivity becomes essential to avoid as many as possible missing cases. However, when sensitivity is prioritised, specificity tends to suffer as a trade-off, with an unavoidable increase in the number of false positive cases needing unnecessary invasive and/or expensive further investigations [33, 34]. This aspect may cause potential physical and psychosocial harms in the subjects attending the screening together with increased healthcare costs, to be considered when evaluating the overall cost-effectiveness of any screening programme [35–37].

Furthermore, in the context of cancer screening, one major drawback of highly sensitive tests lies in the risk of overdiagnosis, which arises when individuals receive diagnoses for conditions that would not become clinically relevant within their lifespan due to their biologically indolent or nonprogressive nature [38]. Interestingly, we should distinguish between “overdetection”, whose responsibility is in the hands of radiologists, and the properly called “overdiagnosis”, whose responsibility should at least be shared with pathologists [39]. Importantly, overdiagnosis can lead to adverse effects for both patients’ well-being and the healthcare system at large, including compromised quality of life and even premature mortality stemming from unnecessary treatments (overtreatment), psychosocial distress due to the inaccurate classification of individuals as patients (stigmatisation), and unwarranted costs derived from the use of healthcare resources for follow-up, treatments, and interventions [40].

Of note, the likelihood of overdiagnosis, as per its definition, is negligible in the context of “diagnostic” tests, i.e. in the clinical scenario. Thus, according to EBM, the decision of whether to administer diagnostic tests should be steered by methodically executed studies on diagnostic performance, i.e. “sensitivity and specificity” studies, involving consecutive patients. These studies should be underpinned by meticulously established clinical decision criteria and dependable reference standards. High-quality and multicentre studies provide the most robust evidence [24].

Conversely, given the heightened risk of overdiagnosis in the screening setting, the EBM principles oriented the European Council Recommendations to state that the introduction of a novel screening tool should only occur after substantiating the clinical relevance on patient outcomes via rigorously conducted RCTs, since mere improvements in sensitivity and specificity are deemed as inadequate to warrant its adoption [41–43].

RCTs remain the most effective approach for tackling two substantial biases that arise within screening programmes, known as “lead bias” and “length bias”. The former entails the inclination to assume greater survival outcomes solely due to the earlier diagnosis, whereas the latter pertains to the phenomenon where slower-growing tumours are more likely to be detected in screening rounds, while faster-growing ones might be missed [44–47]. As a result, focusing solely on the survival of patients who undergo a screening test might create a false impression of improvement, since slower-growing tumours identified during screening tend to stand out more compared to those that go undetected [48].

Nonetheless, the performance of screening tests should be also considered relative to other aspects of cancer management, including the effectiveness of interventions and the availability of facilities for diagnosis and treatment. Indeed, it is crucial in a screening programme to ensure access to treatments that provide advantages when administered at an early stage [49].

## The case for screening mammography for the general average-risk female population

Screening mammography, in particular population-based programmes, have been established in Europe on the basis of RCTs showing clearly favourable results [50]. Only a few European countries still do not have active screening mammography programmes for women aged 50−70 [51, 52]. The Guidelines Development Group of the European Commission Initiative on Breast Cancer [53, 54] confirmed the recommendation to use organised mammography screening for the early detection of BC in asymptomatic women [55].

Indeed, in 2015, the International Agency for Cancer Research published an analysis of data from 20 cohort studies and 20 case-control studies (including RCTs) [56] affirming that women aged 50–69 who participated in mammography screening, experienced a reduction of about 40% in BC mortality. Furthermore, several studies indicated that women aged 70–74 also benefited from a noteworthy decrease in BC mortality through mammography screening. This analysis made a point [57], also regarding potential drawbacks, including the risk of false positive results as well as overdiagnosis, the latter being subject to variable estimates due to different study designs and methodologies [43].

Furthermore, as healthcare interventions evolve over time, so does the role and impact of mammographic screening compared to its initial implementation and to the first trials. This evolution is influenced by the development of more effective novel systemic therapies and heightened BC awareness, underlining the dynamic nature of healthcare strategies. Importantly, even when considering the effectiveness of new target therapies, screening mammography has been shown to still provide important advantages in terms of patient’s outcome [49].

## The case for breast MRI for high-risk screening

As outlined in the Introduction, contrast-enhanced breast MRI has been recognised as a valuable screening tool in women at high BC risk. From 2000 to 2015, several studies reported a large superiority of MRI versus mammography (in some studies also versus ultrasound) in women with hereditary BC predisposition [58–72] (Table 1). Ten studies, 15 papers, over 6000 women enrolled, near 19,000 rounds. The sensitivity ranged 25−58% for mammography, 33−52% for ultrasound, 48−67 for mammography plus ultrasound, and 71−100% for MRI; specificity 93–100%, 91–98%, 89–98%, and 81–98%, respectively. It was a “large” body of evidence, which was already substantial in 2006−2007, when first recommendations were issued. However, those studies were solely intraindividual comparative analyses. This represented the base of evidence for recommending and adopting breast MRI for high-risk screening. No RCT was available.

**Table 1:** Prospective comparative studies on MRI—including screening of asymptomatic women with hereditary predisposition to breast cancer

1^st^ author year [Ref], study name (if any), country	Enrollment (cumulative lifetime risk)	Women analysed	Rounds	Breast cancers detected		Mammography		US		Mammography + US		MRI	
				All (Invasive + DCIS)	Invasive/all	Sens (%)	Spec (%)	Sens (%)	Spec (%)	Sens (%)	Spec (%)	Sens (%)	Spec (%)
Kuhl 2000 [58], Kuhl 2005 [59], Germany	Fam, Mut (≥ 20%)	529	1542	43	34/43 (79%)	33	97	40	91	49	89	91	97
Podo 2002 [60], Sardanelli 2007 [61], Sardanelli 2011 [62]	Fam, Mut (> 30%)	501	1592	52	44/52 (85%)	50	99	52	98	63	98	91	97
Kriege 2004 [63], Rijnsburger 2010 [64]	Fam, Mut (≥ 15%)	2157	8760	97	78/97 (80%)	41	95	NR	NR	NR	NR	71	90
Warner 2004 [65], Canada	Mut (estimated > 40%)	236	457	22	16/22 (73%)	36	100	33	96	NR	NR	77	95
Leach 2005 [66], MARIBS, UK	Fam, Mut (estimated ≥ 30%)	649	1881	35	29/35 (83%)	40	93	NR	NR	NR	NR	77	81
Lehman 2005 [67], USA	Fam, Mut (> 25%)	367	367	4	3/4 (75%)	25	99	NR	NR	NR	NR	100	94
Hagen 2007 [68], Norway	Mut (estimated > 40%)	491	867	25	21/25 (84%)	57^b^	NR	NR	NR	NR	NR	86^b^	NR
Riedl 2007 [69], Riedl 2015 [70], Austria	Fam, Mut (> 20%)	559	1365	40	26/4 (65%)	38	97	38	97	50	96	90	89
Kuhl 2010 [71], EVA Trial, Germany	Fam, Mut (≥ 20%)	687	1679	27	16/27 (59%)	33	99	37	98	48	98	93	98
Trop 2010 [72], Canada	Fam, Mut (> 0%)	184	387	12	9/12 (75%)	58	96	42	94^b^	67	91^b^	83	94
Total/range	Fam, Mut; or Mut (≥ 15%–> 40%)	6360	18,897	357	276/357 (77%)	25–58	93–100	33–52	91–98	48–67	89–98	71–100	81–98

Studies from 2000 to 2015 are listed in order of publication; in case of more than one report (e.g. preliminary, mid-term, final results), the final results are reported

*MRI* magnetic resonance imaging, *DCIS* ductal carcinoma in situ, *Fam* women enrolled for being at elevated familial risk of breast cancer, *Mut* women enrolled for being proven carriers of deleterious mutation in a breast cancer susceptibility gene, *NR* not reported, *Sens* sensitivity, *Spec* specificity, *US* ultrasound

^a^Sensitivities at the time of diagnosis for patients who underwent both mammography and MRI

^b^Biannual (instead of annual) US plus clinical breast examination session

It is interesting to note the wording of the ACS in the 2007 guideline [11]: “Screening MRI is recommended for women with an approximately 20–25% or greater lifetime risk of breast cancer, including women with a strong family history of breast or ovarian cancer and women who were treated for Hodgkin disease.^1^ There are several risk subgroups for which the available data are insufficient to recommend for or against screening, including women with a personal history of breast cancer, carcinoma in situ, atypical hyperplasia, and extremely dense breasts on mammography.” This guideline was issued after considering the results from the first six studies reported in Table 1, performed in Germany, Italy, The Netherlands, Canada, the USA, and the UK, available up until July 2006.

Up to August 26, 2023, according to the Scopus database, this paper [11] got 2122 citations (99^th^ percentile), a number showing its impact on the community of BC specialists. Even though preceded by the NICE recommendations [10], this ACS guideline [11] was a game changer in the history of breast MRI. Thereafter, many other guidelines recommended MRI for screening high-risk women, including those issued by the European Society of Breast Imaging (EUSOBI) in 2008 and 2015 [73, 74], by the multidisciplinary European Society of Breast Cancer Specialists, EUSOMA, in 2010 [75] as well as those issued by the ACR in 2018 and 2023 [76, 77]. The evidence from these comparative studies also showed the erroneous “mantra” about the so-called “low specificity” of breast MRI, which had previously limited the adoption of the new technique [78]. A number of meta-analyses and cost-effective analyses confirmed the diagnostic performances of breast MRI screening in the high-risk setting [79].

Why was breast MRI high-risk screening recommended on the basis of “only” comparative test accuracy studies? Why were the EBM rules not applied? Many reasons can be taken into account [80]. First of all, the undeniable superior accuracy of breast MRI in high-risk women raised ethical concerns that made it difficult to withhold MRI from a control group, especially for solely study purposes [81]: the absolute difference in sensitivity between MRI and mammography considering the ten studies reported in Table 1 ranged from 25 to 60%. Thus, obtaining consent for randomisation was improbable among women with hereditary BC predisposition due to heightened BC awareness, particularly within families with multiple affected individuals.

RCTs assessing the efficacy of MRI for high-risk screening had not been proposed or carried out until 2019, when the results of the first RCT were published by Saadatmand et al. [82], significantly later than the widespread adoption of breast MRI for high-risk screening. From 2011 to 2017, 1355 women provided consent for randomisation (675 allocated to MRI and 680 to mammography group), and 231 women opting for registration (218 to mammography and 13 to MRI). After 4.3 mean rounds/woman, significantly more BCs were detected by MRI (*n* = 40) than by mammography (*n* = 15). The 24 invasive cancers detected by MRI (median size 9 mm) were significantly smaller than the 8 detected by mammography (median size 17 mm) and less frequently node-positive. The stage of BCs detected at incident rounds was significantly earlier and less frequently node-positive in the MRI group than in the mammography group. Of note, all 7-stage ≥ T2 tumours were in the two highest breast density ACR-BI-RADS categories (*c* or *d*).

This trial demonstrated that breast MRI screening can lead to a shift in tumour stage upon detection, thereby reducing the incidence of late-stage cancers, with a consequent decreased need for adjuvant chemotherapy and a reduction in mortality. This is an issue of particular relevance for BRCA mutation carriers due to the rapid growth of cancers and the increased occurrence of triple-negative BCs in these individuals [83, 84]. The role of MRI in the early detection of triple-negative BCs and the effect on survival in high-risk women had been already shown in the Italian HIBCRIT study [85].

Several months after the publication of the work by Saadatmand et al. [82], the German Consortium for Hereditary Breast and Ovarian Cancer published their 10-year experience of high-risk BC surveillance with MRI [16]. In a cohort of 4573 high-risk women (954 BRCA1 carriers, 598 BRCA2 carriers, 3021 BRCA1/2 non-carriers) and 14,142 rounds with MRI between 2006 and 2015, 221 primary BCs (185 invasive, 36 ductal carcinomas in situ [DCIS]) were diagnosed within 12 months of annual screening. Of all cancers, 86% (174/206, 15 unknown) were stage 0 or I. The sensitivity of the programme was 90%, without significant differences by risk level or age. Specificity was significantly lower in the first round (85%) than in subsequent rounds (91%). This experience showed that high-risk screening with MRI could be successfully implemented in clinical practice.

## The case for breast MRI for screening in women with dense breasts

Although breast MRI screening was initially set up to target specific high-risk populations, the extension to women with dense breasts had been already explored in the first experience with contrast-enhanced breast MRI in 1985 [5] and considered as an ongoing issue by the ACS guideline in 2007 [11]. Women with dense breasts were classified as having an “intermediate risk” along with women with previous personal BC or atypical ductal hyperplasia or other lesions with uncertain malignant potential [86]. The results from the DENSE RCT [27] have drastically changed this scenario.

Breast density, which refers to the proportion of fibroglandular tissue in relation to adipose tissue [87], had been identified as an independent risk factor. Women with the densest breasts are four times more likely to develop BC compared to those with predominantly fatty breasts [87, 88]. Moreover, increased breast density masks underlying breast lesions, leading to a reduction in the sensitivity of mammography, dropping from 86–89% in predominantly fatty breasts to 62–68% in extremely dense breasts [89], leading to a high rate of “underdiagnosed” BCs. Thus, breast density represents an accessible variable for risk-adjusted screening strategies in the current era of personalised and precision medicine.

Meanwhile, since the 2000s, digital breast tomosynthesis (DBT) was proposed as a screening tool to overcome the lower mammography sensitivity in the presence of overlapping breast tissues, particularly for specific density and age groups. Cancer detection rates were shown to increase by 20–40% in women with both low- and high-density breast [90, 91], with a differential incremental detection according to breast density, meaning that the increase in cancer detection tends to be greater in high- versus low-density breasts (pooled difference in incremental cancer detection rate of 1.0 per 1000 screens) [92]. However, very limited evidence, if any, of a reduction of interval cancer rate was found [93, 94]. Therefore, DBT does not seem to solve the breast density dilemma.

The results of the DENSE trial deserve the highest attention because this is a RCT, fully compliant with the EBM rules for implementing screening tests. Women aged 50–75 who exhibited extremely dense breast tissue were invited to undergo biennial screening with breast MRI following negative screening mammography [95]. In the first round of screening [27], supplemental breast MRI led to the detection of an additional 16.5 cancers per 1000 screening examinations. A statistically significant and clinically relevant reduction in interval cancer rate versus the control arm was observed both at the intention-to-screen (2.5‰ versus 5.0‰) and at the complier average causal effect analysis (0.8‰ versus 5.1‰), indicating that supplemental MRI screening results in a strong reduction in interval cancer rate, effectively mitigating underdiagnosis. In the subsequent screening round [96], a further MRI detection rate of only 5.8 per 1000 screening examinations was reported, providing evidence that relevant cancers had already been detected by MRI; a reduction in false positive findings was also observed (Table 2).

**Table 2:** Studies on breast MRI screening in women with dense breasts

1^st^ author year [Ref], study name (if any), country	Inclusion criteria	Design/analysis	Participants	Round	Results	Comments
Bakker 2019 [27], DENSE, The Netherlands	Women aged 50−75 with negative mammography and ACR BI-RADS density *d*	Multicentre RCT: MRI *vs* mammography	8061 in the MRI-invitation group (4783 accepted, 59%) 32,312 in the mammography-group	1^st^	Interval cancer rate: 2.5‰ (MRI-group) *vs* 5.0‰ (mammography group) at intention-to-treat analysis; 0.8‰ (MRI group) *vs* 5.1‰ at complier average causal effect analysis	Supplemental MRI screening resulted in the diagnosis of significantly fewer interval cancers compared to mammography alone during a 2-year screening period.
Comstock 2020 [97], EA1141 ECOG-ACRIN, USA, Germany	Women aged 40−75 scheduled to screening with DBT with ACR BI-RADS density *c* or *d*	Multicentre, intraindividual, comparative, cross-sectional study with longitudinal follow-up: abbreviated MRI *vs* DBT	1444 women underwent both examinations		Invasive cancer detection rate: 11.8‰ (MRI) *vs* 4.8‰ (DBT)	Abbreviated breast MRI, compared with DBT, was associated with a significantly higher rate of invasive breast cancer detection.
Veenhuizen 2021 [96], DENSE, The Netherlands	ACR BI-RADS density *d* who already underwent first round of screening with MRI in the DENSE trial	Multicentre RCT: MRI *vs.* mammography	3436 women attending the second MRI screening, out of 4783 attending the first round	2^nd^	Cancer detection rate: 5.8‰ (2^nd^ round) *vs* 16.5‰ (1^st^ round). False-positive rate: 26.3 ‰ (2^nd^ round) *vs* 79.8‰ (1^st^ round). All MRI-detected cancers in the 2^nd^ round were early stage (0–I) and node negative.	The results of 2^nd^ round play in favour of the MRI detection of prevalent cancers at 1^st^ round, not detected by mammography. The availability of prior MRI examination can partly explain the reduction in the false-positive rate.
den Dekker 2021 [98], DENSE, The Netherlands	Positive patients with MRI BI-RADS 3, 4, or 5 years at the first round in the DENSE trial	Multivariable logistic regression	454 women recalled for diagnostic work-up	1^st^	79 true-positives and 375 false-positives. The prediction model based on all clinical characteristics and MRI findings could have prevented 46% of false-positive recalls and 21.3% of benign biopsies without missing any cancers.	Prediction models based on clinical characteristics and MRI findings may be useful to reduce the false-positives first-round screening MRI rate and benign biopsy rate in women with extremely dense breasts.

*ACR BI-RADS* American College of Radiology Breast Imaging Reporting and Data System, *MRI* magnetic resonance imaging, *DBT* digital breast tomosynthesis, *RCT* randomised controlled trial

We acknowledge that these results (i.e. over 80% reduction in interval cancer rate after the first round) are not a direct demonstration of a reduction in disease-specific mortality; however, it can be considered as one of the best proxy parameters of effectiveness of a screening test in the framework of EBM [25].

Interestingly, a further analysis of data from the first round [98] showed that a prediction model based on all clinical characteristics and MRI findings could have prevented 46% of false-positive recalls and 21% of benign biopsies, without missing any cancers.

In addition, we should give relevance to the results of the ECOG-ACRIN EA1141 study [97]. The effectiveness of standalone screening MRI for women with heterogeneously or extremely dense breasts was assessed by comparing abbreviated MRI to DBT, performed with randomised order of execution. Among 1444 women having either heterogeneously dense or extremely dense breasts, breast MRI successfully detected all 17 cases of invasive cancers and identified 5 out of 6 DCIS (83%). In contrast, DBT detected only 7 out of 17 invasive cancers (41%) and 2 out of 6 DCIS (33%). Sensitivity was significantly higher for MRI (96%) than for DBT (39%). Specificity was significantly higher for DBT (97%) than for MRI (87%). The additional imaging recommendation rate was significantly lower for MRI (8%) than for DBT (10%) while the positive predictive value of biopsy at lesion level was higher for DBT (36%) than for MRI (19%), without significant difference.

These study results clearly play in favour of abbreviated MRI screening protocols and show that DBT does not overcome the intrinsic limitations of mammography for screening women with dense breasts, even when a quasi-three-dimensional mammographic technique as DBT is applied. Furthermore, the perspective was enlarged to women with breast density ACR BI-RADS category *c*, considering that 77% of the women analysed had density *c* and only 15% had density *d* (8% had density *b* or even *a*, due to involution after the last screening mammogram). In fact, of the 16 BCs detected by MRI and undetected by DBT, 3 were in density *d*, 11 in density *c*, and 2 in density *b*. No interval cancers were observed during follow-up, but the intraindividual study design does not allow comparing the interval cancer rate for each of the two screening methods.

A recent systematic review and meta-analysis including 22 studies [99] reported that of 132,166 screened women with dense breasts and negative mammography, a total of 541 cancers missed at mammography were detected with supplemental modalities, including DBT, handheld or automated breast ultrasound, and MRI. MRI was significantly superior to the other modalities in cancer detection rate (1.52 per 1000 screenings), including invasive cancers (1.31 per 1000 screenings) and DCIS (1.91 per 1000 screenings), without significant differences in recall or biopsy rate. The authors highlight that the limited number of studies prevented assessment of interval cancer rates. Excluding MRI, no significant difference in any metrics was identified among the remaining modalities.

The integration of breast MRI for screening women with dense breasts into practice, despite its reported potential benefits (supported by comparative studies and finally substantiated by a RCT), has encountered several challenges. A prominent obstacle is the considerable cost associated with MRI screening, which includes not only the imaging itself but also the specialised equipment and personnel required [100]. A further practical problem is the request for more additional tests. These implications can place significant strains on healthcare systems, hampering their capacity to extend routine MRI screenings to a broader population beyond the subgroup of high-risk women. Presently, ultrasonography is a more common choice for supplemental screening thanks to its broader availability and lower implementation costs, despite its modest additional cancer detection rate, as also shown by the above-mentioned meta-analysis [99]. However, a risk-adjusted strategy could potentially optimise resource allocation [101, 102]: based on data from the DENSE trial, MRI alone every 4 years in women with extremely dense breasts is cost-effective with € 15,620 per quality-adjusted life years.

In this scenario, the most updated guidelines from the European Commission Initiative on Breast Cancer (ECIBC) [103] propose the following suggestions regarding asymptomatic women with high breast density 45−74 years old in the context of an organised population-based screening programme:

- Not implement tailored MRI screening after a negative mammogram (issued in January 2020).
- If high mammographic breast density is detected for the first time with digital mammography, implement tailored screening with DBT in the next screening round (issued in September 2021).

The update on MRI screening was undertaken a few months after the publication of the DENSE trial results and before the publication of the ECOG-ACRIN EA1141 study as well as of analyses and models about cost-effectiveness and possible prolonged intervals between MRIs. Of note, the ECIBC Guidelines Development Group defined this recommendation as “conditional” and with a “very low certainty of the evidence”. In addition, almost four years after issuing the guideline, the proposed research priorities (balance of effects, including the potential risk of adverse events due to contrast reaction; to improve the specificity of MRI-tailored screening; to study abbreviated protocols to make the intervention less costly and more acceptable) can be considered substantially solved. Nowadays, the recommendation in favour of DBT (which applies to women with both density *c* or *d*) appears taking into consideration more practical feasibility than the evidence available. It remains to see what the next ECIBC guidelines update will propose.

To summarise, despite level 1 evidence in favour of breast MRI screening of women with dense breasts, the practical challenges related to costs, availability, and additional assessments have prevented real adoption of this screening modality, even for the limited group of women with extremely dense breasts. An overall comparison between the two cases of breast MRI screening for women at high risk and for women with dense breasts is provided in Table 3.

**Table 3:** Breast MRI screening: available evidence, recommendations, and adoption in women at high risk and in women with dense breasts

Available evidence, recommendations/guidelines, and adoption	Breast MRI screening	
	High risk	Dense breasts
RCT: mortality reduction	No	No
RCT: interval cancer reduction	No	Yes (in 2019)^a,c^
RCT: favourable shift in tumour stage upon detection	Yes, in 2019^d^	Yes (in 2019)^a,c,e^
Test accuracy comparative studies	Yes, since 2000^f^	Yes (in 2020)^b,g^
Recommendations/guidelines	Since 2006−2007^h,i^	2018−2023^j, k^
Adoption by healthcare systems	Yes	No

*MRI* magnetic resonance imaging, *RCT* randomised controlled trial

^a^Extremely dense breasts (BI-RADS category *d*)

^b^Heterogeneosly dense and extremely dense breasts (BI-RADS categories *c* and *d*)

^c^DENSE, main results 1^st^ round [27]

^d^FaMRIsc [82]

^e^DENSE, second round [96]

^f^Ref [58]

^g^ECOG-ACRIN EA1141 [97]

^h^NICE [23]

^i^ACS [11]

^j^ACR 2018 [76]

^k^ACR 2023 [77]

## Conclusion: a double difference between theory and practice

When comparing breast MRI screening in high-risk women and in women with extremely dense breasts, we appreciate a difference between the EBM theory and real-world practice. The difference in accuracy and BC detection in favour of MRI in high-risk women was so large that the new test was adopted regardless of the lack of results from RCTs. On the contrary, despite having positive results from an RCT, lack of resources does not allow implementation of breast MRI screening for women with extremely dense breasts. Women with extremely dense breasts are estimated to be on average 10% of the female population from 50 to 70 years of age [28], which can be translated into about 6−7 million in the European Union [104] (much more than those at high-risk, considering that for example, the prevalence of BRCA1 and BRCA2 mutation carriers in the general population is around 0.2% [105]). Health authorities in The Netherlands suggested an alternative way: contrast-enhanced mammography [106, 107] instead of MRI, due to its lower cost and higher accessibility [108]. Trials are ongoing, such as the C-MERIT [109] and the CMIST [110], but we need their results before adopting this solution.

The current era of big data is fostering a new way of thinking about the relation between RCTs and real-world evidence: a more fruitful interplay between them is expected [111, 112]. However, when we look at the gap between EBM theory and breast MRI screening, a sentence attributed to Manfred Eigen, 1967 Nobel Laureate in Chemistry, seems to be appropriate: “In theory, there is no difference between theory and practice. But in practice, there is”.

## Abbreviations

ACR: American College of Radiology
ACS: American Cancer Society
BC: Breast cancer
BI-RADS: Breast Imaging Reporting and Data System
DBT: Digital breast tomosynthesis
DCIS: Ductal carcinoma in situ
EBM: Evidence-based medicine
MRI: Magnetic resonance imaging
NICE: National Institute for Clinical Excellence
NMR: Nuclear magnetic resonance
RCT: Randomised controlled trial

## Footnotes

1 The ACS recommended breast MRI “as an adjunct to mammography”: (1) for women at familial/genetic high risk (BRCA-mutated women, their untested first-degree relatives, or with estimated lifetime risk ≥ 20–25%) on the basis of nonrandomized comparative studies, (2) for women who had radiation to chest between age 10 and 30 years on the only basis of estimated lifetime risk. The latter approach was used also for Li-Fraumeni syndrome and first-degree relatives as well as Cowden and Bannayan-Riley-Ruvalcaba syndromes and first-degree relatives. Of note, breast MRI screening recommendations on the only basis of lifetime risk estimates, without any supporting MRI study, were even more challenging the EBM theory than those based on test accuracy comparative studies.
